# Valve Replacement for Massive Hemoptysis in Mitral Stenosis: An Uncommon Course in Modern Practice

**DOI:** 10.7759/cureus.13960

**Published:** 2021-03-17

**Authors:** Adil H Al-Kindi, B Jayakrishnan, Hatim Al-Lawati, Saif Al-Mubaihsi, Suresh Chengode

**Affiliations:** 1 Department of Surgery, Sultan Qaboos University Hospital, Muscat, OMN; 2 Department of Medicine, Sultan Qaboos University Hospital, Muscat, OMN; 3 Department of Anesthesia, Sultan Qaboos University Hospital, Muscat, OMN

**Keywords:** hemoptysis, mitral stenosis, mitral valve replacement

## Abstract

Massive hemoptysis is uncommon in mitral stenosis in contemporary practice. We report a patient without any previous illness presenting with life-threatening pulmonary hemorrhage, who was initially managed as cryptogenic hemoptysis. Once mitral stenosis was confirmed, the patient underwent mitral valve replacement with total and complete cessation of bleeding.

## Introduction

Massive hemoptysis has been defined in various studies as expectoration of 100 to 1000 ml of blood over a wide-ranging period of time [1]. This may lead to asphyxia and could turn fatal as the anatomical dead space of the major airways is only 100 to 200 ml [2]. There are several causes for massive hemoptysis, ranging from the common ones like bronchiectasis, tuberculosis, bronchogenic carcinoma to uncommon ones like Hughes Stovin syndrome, angioinvasive fungal infections, or Dieulafoy’s disease [3-6]. Hemoptysis is known to occur in mitral stenosis frequently, but massive pulmonary hemorrhage is uncommon. Cases have been reported as early as 1930s [7]. Earlier studies estimate a high incidence of hemoptysis in mitral stenosis ranging from 9.5% to 42.5% [8]. However, hemoptysis due to mitral stenosis is rare in contemporary practice because of early recognition and intervention, and its presence usually suggests an advanced disease [9].

## Case presentation

A 53-year-old obese truck driver who was a heavy smoker presented to the emergency of Sultan Qaboos University Hospital, Muscat, Oman with massive hemoptysis. He never had any major symptoms or hemoptysis before. When assessed, he was coughing and retching at the same time, and it was difficult to determine whether it was hemoptysis or hematemesis. The patient was afebrile and had a blood pressure of 170/90 mm Hg, heart rate of 112 beats/min, arterial oxygen saturation of 91% on room air, and respiratory rate of 20 breaths/min. Initial laboratory studies showed the following results: hemoglobin, 13.0 g/dL; leukocyte count, 15.3 x10^9^/L; platelet count, 281 x10^9^/L; aspartate aminotransferase, 46 U/L; and alanine aminotransferase, 63 U/L. The patient’s coagulation profile on admission was normal specifically, the international normalized ratio (INR), 1.04; prothrombin time, 11.3 seconds; activated partial thromboplastin time (APTT), 30.0 seconds; thrombin time=16.7 seconds and fibrinogen=3.3 g/L.

**Figure 1:**
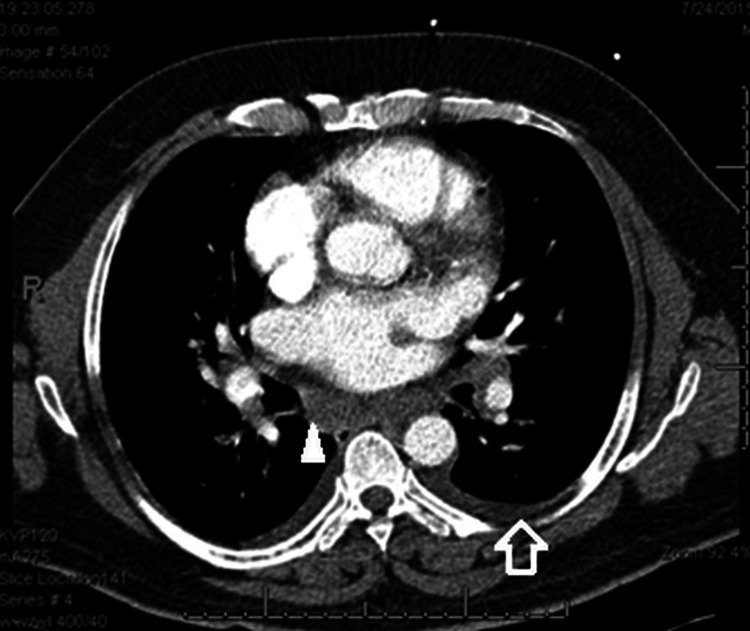
Computed tomography (CT) of the chest showing bilateral pleural effusions (open arrow) and mediastinal adenopathy (arrow head).

**Figure 2:**
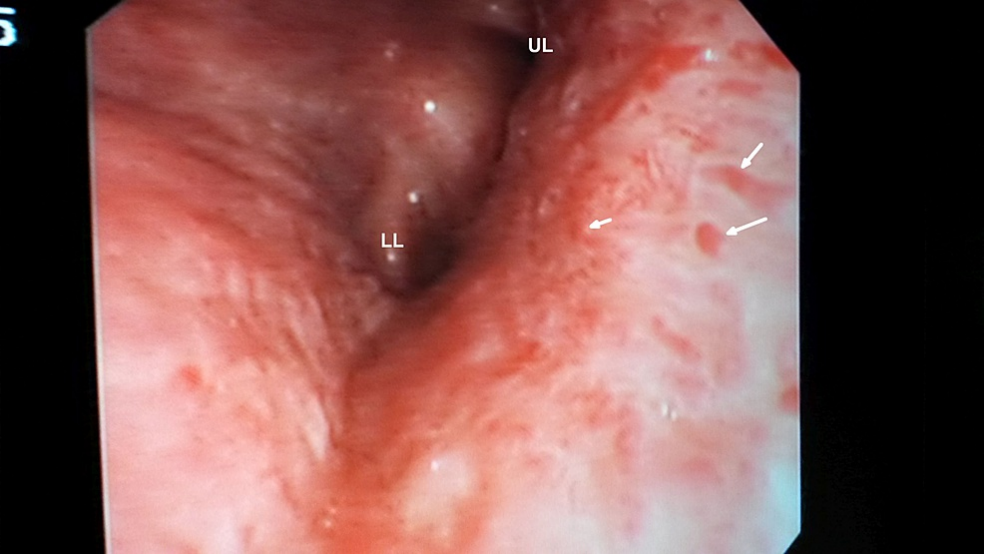
Slightly edematous mucosa of the left main bronchus, small nodular elevations (white arrows) and high vascularity. UL; Upper lobe, LL; Lower lobe

Chest radiograph showed haziness of both lower zones with prominent hilar shadows bilaterally. Computed tomography (CT) revealed bilateral minimal pleural effusions, mediastinal adenopathy, slight haziness in the left lower zone with a few nodular areas, and narrowing of the left lower lobe bronchus (Figure 1). A bedside echocardiogram, difficult because of poor acoustic windows, showed normal bi-ventricular function. Fiberoptic bronchoscopy showed pooling of blood in the airway, a bulge in the lower part of trachea, blunting of carina, and focal vascular areas with small nodular elevations (Figure 2). Endobronchial biopsy was done from the lower tracheal lesions. Upper gastrointestinal endoscopy revealed blood in the stomach and duodenum with red spots and streaks in the gastric mucosa. During the fiberoptic bronchoscopy also he continued to cough out large quantities of blood, around 600 ml in one hour. The patient was intubated to complete the endoscopies.

Bronchial arteriogram showed normal anatomy and no aneurysms, dilatations, or bleeding areas. Bronchial and gastric biopsies did not give any additional information. Methylprednisolone was started suspecting vasculitis, supported by a finding of a few eosinophils in the bronchial biopsy and lack of definite lesions on bronchoscopy. Mediastinoscopy was done to sample the lymph nodes. The patient developed massive hemoptysis again the very next day with oxygen saturation dropping down to 44%. The patient was reintubated, sedated, and paralyzed to suppress the intermittent coughing. Bleeding could be controlled only with the administration of recombinant activated factor VII (rFVIIa).

No definitive etiology or a culprit anatomical location in the lung was identified up to this point. Pulmonary parenchyma as seen in the CT was not showing consolidations, infiltrates, or cavities. Hemoptysis persisted while all the blood works, immunologic screening, bronchoscopy, mediastinal lymph node biopsy, and a screening echocardiogram failed to identify the cause. Bronchial artery embolization was not considered as the angiogram did not reveal any abnormality. A multidisciplinary meeting and reassessment of the case was done at this point. 

**Figure 3:**
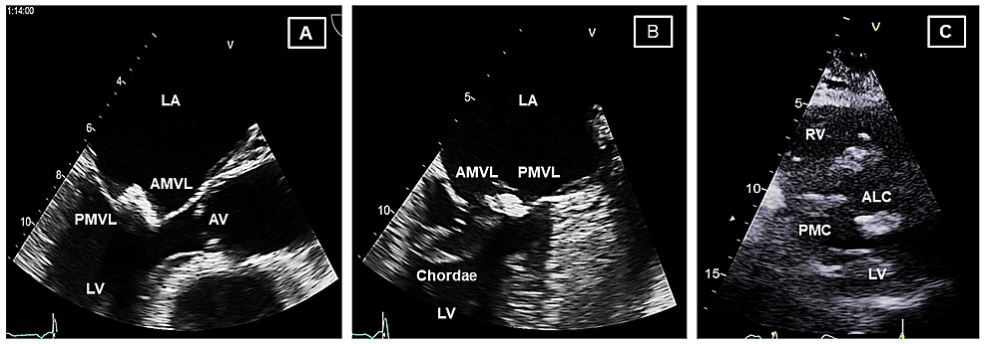
Representative images from the transesophageal (A, B) and transthoracic (C) echocardiographic studies showing severely calcific and stenotic mitral valve. (A) Mid-esophageal long axis view (120°); dense calcification involving the tips of the anterior and posterior mitral valve leaflets. Mitral valve motion is severely restricted. (B) Mid-esophageal two chamber view (90°); dense calcification is again noted to involve the tips of the anterior and posterior mitral leaflets and marked thickening of the chordal apparatus). (C) The parasternal short axis view at the level of the mitral valve showing marked calcification at the anterolateral and posteromedial commissures. ALC; anterolateral commissure, AMVL; anterior mitral valve leaflet, LA; left atrium, LV; left ventricle, PMC; posteromedial commissure, PMVL; posterior mitral valve leaflet. RV: right ventricle

Mitral valve calcification was suspected when the CT was reevaluated. A comprehensive departmental trans-thoracic echocardiogram showed calcified rheumatic mitral valve with doming of the anterior leaflet and a calcified mass freely moving at the tip. There was considerable bi-commissural calcification (Figure 3). Trans-esophageal echocardiogram confirmed severe mitral stenosis with a mean gradient of 13 mmHg across the mitral valve and a mitral valve area (MVA) of 0.7cm^2.^, calculated using the pressure half-time method. The heart rate was 77 beats per minute and the patient was in sinus rhythm. Coronary angiogram showed normal coronaries. Right heart catheterization showed severe rheumatic mitral stenosis (MVA= 0.73 cm^2^, Mean gradient= 20.7 mmHg), severe pulmonary hypertension with near systemic pressures, and pulmonary vascular remodeling. Systolic pulmonary artery (PA) pressure was 103 mmHg; mean pulmonary artery (PA) pressure, 68 mmHg, left ventricular end diastolic pressure (LVEDP), 16 mmHg; mean pulmonary capillary wedge pressure (PCWP), 39 mmHg; trans pulmonary gradient (TPG), 29 mmHg and right atrial (15 mmHg) and left atrial (39 mmHg) pressures were elevated. The high TPG in our patient without any significant lung disease indicates that pulmonary hypertension is not purely due to left-sided heart disease and suggests pulmonary vascular disease secondary to long-standing and untreated left ventricular inflow obstruction. Furthermore, the computed pulmonary vascular resistance of ~12 woods units confirmed significant pulmonary vascular remodeling.

The morphology of the mitral valve on echocardiography was unfavorable for percutaneous mitral balloon valvotomy. The patient was, therefore, taken for an emergent mitral valve surgery. On exposure of the valve, there was severe stenosis of the mitral valve orifice. The valve was markedly thickened with severe calcification and calcium chunks. The pathology was extending down to the papillary muscles. The valve was then resected with the preservation of sub-valvular apparatus. The papillary muscles were re-suspended to the annulus. Mitral valve replacement was done with 25/33 On-X mechanical valve (Cryolife Inc., GA, USA). He was subsequently weaned from cardiopulmonary bypass on moderate inotropic support.

Post-operative recovery was slow due to the development of severe sensory-motor axonal neuropathy. He was anticoagulated with warfarin for a target INR of 2.5-3.0. There was no recurrence of hemoptysis in the hospital or after discharge.

## Discussion

We report a patient with severe mitral stenosis who presented for the first time with massive hemoptysis. Absence of prior symptoms in spite of a job needing heavy physical exertion and a normal screening echocardiogram delayed the diagnosis. Massive pulmonary hemorrhage as a rare presenting symptom of mitral stenosis has been reported [10,11]. When massive, a pulmonary pathology is suspected and the association of hemoptysis with mitral stenosis is often overlooked [12].

Massive hemoptysis can turn potentially fatal as this can often lead to asphyxia [13]. Ideally, life-threatening hemoptysis should be judged by the total volume and speed of the bleeding as well as the patient’s cardiopulmonary reserve. In nearly 20% of cases, the cause of hemoptysis remains unknown [4]. Since the initial extensive workup was inconclusive, pulse methylprednisolone was given for possible vasculitis. Moreover, we had to administer rFVIIa as a salvage therapy. Isolated case reports of the potential efficacy of recombinant activated factor VII have been described in patients with pulmonary hemorrhage due to cystic fibrosis, pneumonia, trauma, thrombocytopenia, bone marrow transplantation, and vasculitis [14].

Hemoptysis in patients with mitral stenosis is rare these days because of early diagnosis and intervention. Mitral stenosis has been reported to cause five different forms of hemoptysis: pulmonary apoplexy, congestive hemoptysis, pulmonary edema, winter bronchitis, and pulmonary infarction [15]. Hemoptysis is attributed to sudden dilatation of pulmonary capillaries and diapedesis of red blood cells into pulmonary alveoli, rupture of necrotic vessels involved in acute rheumatic process, shunting of blood from pulmonary to bronchial veins, or bleeding from the pleurohilar veins [8,13]. In our patient, the pulmonary pressures reached near systemic pressures and so the bleeding would have occurred from the pulmonary circulation across both the lungs. Wood described this as pulmonary apoplexy, a sudden profuse hemoptysis often antedating the onset of dyspnea, probably due to abrupt increase in pulmonary venous pressure before the development of pulmonary hypertension [15]. In that series, apoplexy was the cause of hemoptysis in 18.3% of cases, 42.5% being the first manifestation. Though Wood believed that pulmonary apoplexy is a benign self-limiting condition with hemorrhage serving to decompress the bronchial system, the hemorrhage being massive can turn fatal due to the chances of asphyxia.

Symptoms, the most common being dyspnea, stem from impaired blood flow across the mitral valve and pressure overload in pulmonary circulation which becomes apparent when the valve area is ≤ 2.5 cm^2^ [9]. The most common presenting symptom is dyspnea on exertion, caused by a decreased capacity to increase cardiac output during exertion or raised left atrial and pulmonary venous pressures leading to pulmonary edema [9]. With chronically elevated left heart pressure, bronchial mucosa may become hyperemic with dilatation of the sub mucosal veins due to a reverse flow of blood from the pulmonary to the bronchial venous plexus. Dilatation of the bronchial vasculature may be the first hint of elevated left atrial pressure [16]. This was seen in bronchoscopy in our patient, but was not recognized as they were focal and distributed unevenly

Balloon valvotomy can relieve obstruction and provide immediate relief. However, it cannot be used if there is concomitant moderate or severe mitral regurgitation, the valve apparatus is too fibrotic or calcified, or there is a left atrial thrombus. Mitral valve surgery is associated with rapid and sustained cessation of pulmonary hemorrhage [17-19]. Successful mitral commissurotomy for severe hemoptysis has been reported as early as the 1950s [20]. Successful mitral valve replacement will result in consistent decrease in pulmonary vascular resistance despite an increase in blood flow whereas after a closed commissurotomy pulmonary resistance may decline, increase or remain unchanged [12].

## Conclusions

Our patient's presentation demonstrates that mitral stenosis should still be considered in the differential diagnosis of severe hemoptysis in contemporary practice. Emergency mitral valve replacement controlled the hemoptysis, presumably by rapid reduction in left atrial pressure and decreased flow through the bronchial venous system.
